# Carbon ion radiotherapy optimization techniques for pancreatic cancer: accounting for the effect of bowel gas variation

**DOI:** 10.1007/s00066-025-02450-8

**Published:** 2025-08-21

**Authors:** Chaebeom Sheen, Sunghyun Lee, Bitbyeol Kim, Jaeman Son, Kyungsu Kim, Hyeongmin Jin

**Affiliations:** 1 Seoul National University College of Medicine, 101 Daehak-ro, Jongno-gu, Seoul, 03080, Korea (Republic of); 2 Project Group of the Gijang Heavy Ion Medical Accelerator, Seoul National University Hospital, 101 Daehak-ro, Jongno-gu, Seoul, 03080, Korea (Republic of); 3 Department of Radiation Oncology, Seoul National University Hospital, 101 Daehak-ro, Jongno-gu, Seoul, 03080, Korea (Republic of); 4 Department of Radiation Oncology, Seoul National University College of Medicine, 101 Daehak-ro, Jongno-gu, Seoul, 03080, Korea (Republic of); 5 Institute of Radiation Medicine, Seoul National University Medical Research Center, 101 Daehak-ro, Jongno-gu, Seoul, 03080, Korea (Republic of)

**Keywords:** Particle therapy, Locally advanced pancreatic cancer, Robust treatment planning, Air cavity, Density-override optimization

## Abstract

**Background:**

Dose variation due to changes in bowel air poses significant challenges for carbon radiotherapy in pancreatic cancer. This retrospective study evaluated a density-override optimization technique to mitigate dosimetric uncertainties caused by bowel air changes.

**Materials and methods:**

Planning CT and cone-beam CT data from 8 patients with locally advanced pancreatic cancer undergoing stereotactic ablative radiotherapy were analyzed. Treatment simulations used a dose of 55.2 GyE in 12 fractions with a four-field setup (anterior, lateral, posterior, posterior oblique). Four density-override patterns were compared: pattern 0 (no override), pattern 1 (replacing bowel gas with water), pattern 2 (replacing the entire bowel with mean bowel HU), and pattern 3 (replacing bowel gas with mean bowel HU). Dose evaluations included fraction-wise and accumulated dose analyses, focusing on target coverage, homogeneity index, and organs at risk doses.

**Results:**

Pattern 2 achieved the largest clinical tumor volume coverage and the fewest fractions with > 5% coverage loss for the anterior beam, followed by pattern 3. However, pattern 2 demonstrated poorer homogeneity for the lateral beam compared to patterns 1 and 3 and a higher gastrointestinal (GI) dose for the anterior beam.

**Conclusion:**

This study evaluated the importance of density overrides to address bowel air variations. For patients where a more uniform dose is desirable or whose tumor is adjacent to the GI tract, a pattern 3 density-override should be considered.

## Introduction

Pancreatic cancer is a malignancy characterized by increasing incidence and high mortality. In the US in 2021, the incidence was reported to increase at a rate of 0.5%–1.0% per year. Most pancreatic cancer patients are diagnosed in either a metastatic (50%) or a locally advanced (30–35%) state unamenable to surgery [1]. The prognosis for locally advanced patients is poor, with Hammel et al. reporting in an international randomized clinical trial that median overall survival (OS) was 16.5 months in locally advanced pancreatic cancer (LAPC) patients who were treated with chemotherapy alone [2]. Current National Comprehensive Cancer Network (NCCN) guidelines recommend chemotherapy alone, stereotactic ablative radiotherapy (SABR), or chemoradiotherapy as neoadjuvant treatment for LAPC [3]. An emerging therapy that could potentially replace traditional photon-based radiation therapy is particle radiotherapy, including carbon ion radiotherapy (CIRT).

The advantages of CIRT are based on both its dosimetric properties and high relative biological effectiveness (RBE). The dosimetric advantages of CIRT arise from the Bragg peak, where most of the energy is deposited. This allows for a spread out Bragg peak (SOBP), where most of the energy is conformally delivered to the gross tumor volume, with improved sparing of organs at risk (OAR) [4]. Meanwhile, the high RBE arises from high linear energy transfer (LET). Thus, CIRT is less dependent on re-assortment within the cell cycle and oxygenation of the tumor. Furthermore, it can have several auxiliary effects such as anti-angiogenic properties and immune adjuvant effects [5].

Due to the low oxygen enhancement ratio, CIRT is expected to have high efficacy in pancreatic cancer, which is traditionally considered hypoxic and radioresistant [6]. While phase III randomized clinical trials comparing CIRT to conventional photon-based radiotherapy are pending, several clinical studies have demonstrated the advantages of CIRT. The J‑CROS 1403 study by Kawashiro et al., which was a retrospective analysis of 72 LAPC patients from three institutions, administered a dose of 52.8–55.2 GyE in 12 fractions. Median OS was shown to be 21.5 months, while 2‑year OS was 46%. These results were superior to the outcomes reported by Hammel et al., where median OS was 15.2 and 16.5 months, respectively, with chemoradiotherapy and chemotherapy alone [7].

However, it is known that variations in bowel gas can cause significant dosimetric changes in carbon ion radiotherapy of pancreatic cancer. Kumagai et al. demonstrated that intrafractional bowel gas bubble movement within a 150‑s timeframe, either due to peristalsis or respiratory motion, degraded target coverage and increased the dose to OAR [8].

Therefore, a robust CIRT treatment plan in pancreatic cancer should account for gastrointestinal gas variation. Clinical guidelines have not yet been established regarding how to account for differential bowel filling. A common methodology employed is to virtually overwrite bowel contents in treatment planning to account for interfractional variations. Kusano et al. compared three patterns of virtually overwriting the bowel. The first involved replacing the bowel gas with a region with no gas. The second overrode the entire bowel with its mean relative stopping power ratio (rSPR; calculated relative to water). No override was performed in the third pattern. It was shown that the second pattern showed the least amount of interfractional clinical target volume (CTV) coverage variation. However, the study did not assess the impact of each pattern on the dose to OAR.

In this retrospective study, we quantify the impact of bowel gas variation during CIRT of LAPC and compare the mitigating strategies that might be employed to increase robustness against gas variation. Specifically, we evaluate four replacement patterns: pattern 0 (no replacement), pattern 1 (replacement of bowel gas with water-equivalent density), pattern 2 (replacement of the entire bowel with a homogeneous mean density), and pattern 3 (replacement of bowel gas with the same mean density used in pattern 2). While patterns 0 and 1 follow approaches previously proposed by Kusano et al., patterns 2 and 3 are original strategies developed to be compatible with current clinical workflows. A focus was set on the dose deposition in OAR as well as CTV coverage to assess how effectively the benefits of CIRT can be realized in clinical practice.

## Materials and methods

### Study population

We retrospectively analyzed data from patients with LAPC who were treated with SABR at the Seoul National University Hospital, Department of Radiation Oncology. This study was conducted in accordance with the principles of the Declaration of Helsinki with the approval of the institutional review board of our institution (2312-094-1492; March 13, 2024). Eight patients who underwent SABR at a dose of 50 Gy in 5 fractions between January 2016 and October 2023 were included in the study. Cone-beam computed tomography (CBCT) was taken for each fraction. All planning CT (pCT) and CBCT images were taken in a head-first supine (HFS) position with arms over head, with a pneumatic abdominal compressor to decrease intra-fractional respiratory tumor movement. All patients fasted for at least 6 h before the pCT and each fraction of SABR [9].

Inclusion criteria for the study included: (1) patients with pathologically or cytologically confirmed invasive ductal adenocarcinoma of the pancreas, (2) patients with radiologically unresectable primary pancreatic tumors classified as T4 based on the seventh edition of the American Joint Committee on Cancer (AJCC) TNM staging system [10], (3) absence of distant metastasis or peritoneal seeding, and (4) Eastern Cooperative Oncology Group (ECOG) performance status of 0 to 2. Exclusion criteria for the study included (1) patients who had been previously operated for primary tumor; (2) patients who had previously been treated with radiotherapy for primary tumor; (3) patients who had metal stent placed near the hepatobiliary region for the alleviation of obstructive jaundice [7, 11]; and (4) patients in whom the distance of the contoured gross tumor volume (GTV) from the duodenum, stomach, and bowel was suggestive of involvement of the bowel wall. This was judged to be true when the distance from the GTV and the duodenum, stomach, and bowel was below 5 mm [12]. A summary of the patient data is available in Table 1.

**Table 1:** Patient characteristics.

Patient	Sex	Age	Loc	Performance	CA 19‑9 (U/ml)	CTV volume (cc)	PTV volume (cc)
Case 1	M	73	Head	ECOG 1	46	118.90	168.22
Case 2	M	73	Body	ECOG 1	73	109.96	157.19
Case 3	F	51	Head	ECOG 1	< 2	119.95	167.40
Case 4	M	68	Body	ECOG 0	25	123.81	176.42
Case 5	F	65	Neck	ECOG 0	12	97.80	143.07
Case 6	F	41	Body	ECOG 0	19	118.63	163.94
Case 7	M	51	Head	ECOG 1	11	115.31	168.61
Case 8	M	77	Tail	ECOG 0	32	77.07	120.63

*Loc* anatomic location of the tumor within the pancreas, *ECOG* Eastern Cooperative Oncology Group, *CA19-9:* carbohydrate antigen 19‑9, *CTV* clinical target volume, *PTV* planning target volume

### Replacement pattern

We used the density-override method, in which the mass density relative to water (relative mass density, RMD) of specific regions of interest (ROI) within the patient were overwritten using the treatment planning system (TPS) of RayStation 2023 B R (RaySearch Laboratories AB, Stockholm, Sweden). To implement the replacement value, the bowel gas (or G‑gas) for each planning CT was delineated. The bowel gas was first delineated using thresholding in the contouring software (Eclipse 16.1; Varian Medical Systems, Palo Alto, CA, USA), with a threshold of −500 Hounsfield units (HU). The thresholding results were then manually revised along the expected path of the beams that lay across the longitudinal length of the planning target volume (PTV). The resulting bowel gas contours were approved by a radiation oncologist with more than 10 years of experience. The following four patterns were studied:

#### Pattern 0 (no replacement)

Optimization of the dose distribution was performed without any replacement. This is identical to the third pattern proposed by Kusano et al. where no replacement was performed [13].

#### Pattern 1 (G-gas to water)

**Fig. 1:**
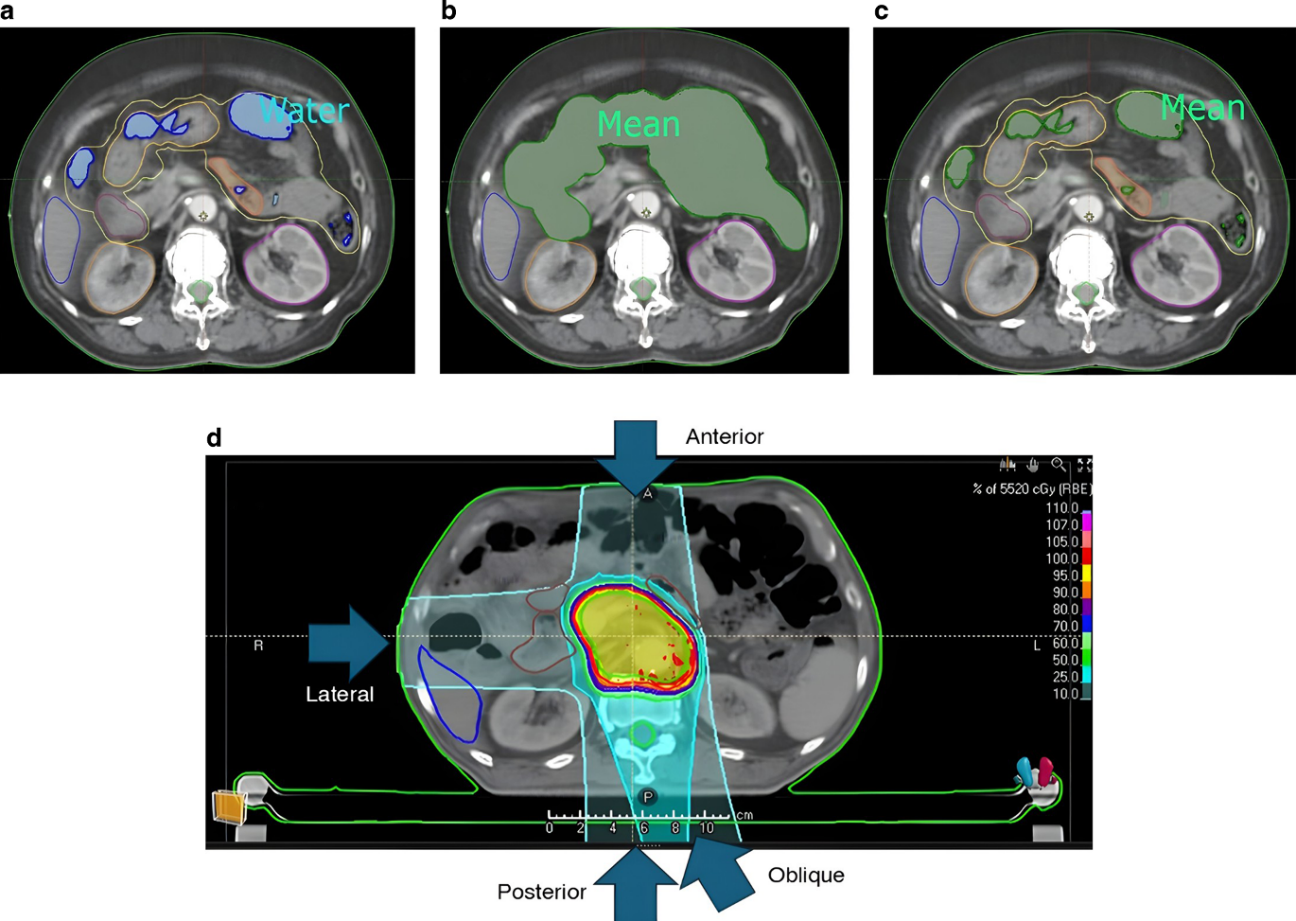
Replacement patterns 1, 2, and 3. In pattern 1 (**a**), the density value of bowel gas is overwritten with the relative mass density of water, i.e., 1.0. Meanwhile, in pattern 2 (**b**), the density value of the whole bowel (area in green) is overwritten to be G_mean_ (mean Hounsfield unit of entire bowel). In pattern 3 (**c**), the density value of bowel gas (area in green) is overwritten to be G_mean_ (mean Hounsfield unit of entire bowel). An example of a treatment plan for pattern 0 (no replacement) is shown in (**d**). In this example, the gantry angles were set to anterior (0 °), lateral (270 °), posterior (180 °), and oblique (165 °) directions

The ROI was set as the bowel gas along the gastrointestinal tract. The RMD of the bowel air region was overwritten to be equal to that of water (which is 1) using the values provided by the TPS. This is identical to the first pattern proposed by Kusano et al. where it is assumed that no air is in the gastrointestinal tract ([13]; Fig. 1a).

#### Pattern 2 (bowel to G_mean_)

The ROI was set as the entire bowel. The RMD of the whole bowel was set as a homogeneous material, whose RMD was determined using the mean HU of the entire bowel, including the gaseous regions, with soft tissue as the reference material. The mean HU and corresponding RMD values are available in Table 2. This method of replacement bears a semblance to the second pattern proposed by Kusano et al. However, Kusano et al. subdivided the bowel region into the upper-middle stomach, lower stomach, first–second part of the duodenum, third part of the duodenum, fourth part of the duodenum, small intestine, right colon, and left colon [13]. We used a coarser pattern of replacement where the entire bowel was replaced, which is less precise but aligns better with existing clinical workflows (Fig. 1b).

**Table 2:** Summary of the relative mass density of the entire bowel for each patient. For patterns 2 and 3, the entire bowel and gas, respectively, were overwritten to a homogeneous material with the corresponding relative mass density.

Patient	RMD	Average HU
Case 1	0.96	−88.15
Case 2	0.92	−121.87
Case 3	0.99	−42.24
Case 4	0.90	−135.63
Case 5	1.01	−4.87
Case 6	1.02	6.17
Case 7	0.95	−89.51
Case 8	0.87	−165.03

*RMD* relative mass density; *HU* Hounsfield unit

#### Pattern 3 (G-gas to G_mean_)

The ROI was set as the bowel gas along the gastrointestinal tract. The RMD of the bowel air region was overwritten to be the RMD corresponding to the mean HU of the entire bowel, including the gaseous regions, with soft tissue as the reference material. Thus, the replacement RMD used in the density override was identical to the one used in pattern 2. This method of replacement uses a less proactive region of replacement compared to pattern 2 to better respect existing imaging for the GI tract (Fig. 1c).

The mean CT attenuation of the bowel in terms of HU was converted to RMD using a linear equation provided by the TPS, assuming that all pCTs were generic CTs.

### Treatment planning

The gantry angles were set to posterior (180°), anterior (0°), lateral, and posterior oblique (oblique) directions, with the patient in the HFS position. The lateral direction was adjusted between either the right (270°) or the left (90°), depending on which area had the least amount of gas. The posterior oblique beam was similarly adjusted to between 150°, 165°, 195°, and 210°, depending on the arrangement of the OAR and the bowel air (Fig. 1d).

Treatment plans were prepared at a dose of 55.2 GyE in 12 fractions with an equal dose for each fraction (4.6 GyE × 12). Three fractions were delivered for each gantry angle. To minimize normal tissue toxicity, the dose constraints of the gastrointestinal tract (GI), spinal cord, and kidney were considered. The minimum doses to the most exposed 2 cm^3^ ($D_{2\mathrm{cc}}$) and 6 cm^3^ ($D_{6\mathrm{cc}}$) of the GI structures were restricted to 46 GyE and 30 GyE, respectively. The maximum dose ($D_{\max }$) to the spinal cord was limited to 30 GyE. The volume of each kidney that received at least 15 GyE was limited to under 30% of the total kidney volume. The treatment plans were made to cover 95% of the CTV and 90% of the PTV with at least 95% of the prescribed dose [7, 13, 14]. Each beam, calculated as a beam applied using the pencil scanning technique, was optimized separately, in line with the single field uniform dose (SFUD) method typically used to treat pancreatic cancer. Scanning was performed using a square grid with spot spacing of 2.5 mm and an energy layer spacing of 3.0 mm water-equivalent thickness. Treatment planning was performed using the RayStation 2023 B R TPS. The GyE dose was calculated based on the microdosimetric kinetic model (MKM), a biophysical model widely used in carbon ion radiotherapy to estimate the RBE-weighted dose.

### Evaluation of dosimetry

The artifacts of CBCT images are well known, including noise, scattering, beam-hardening artifacts, and aliasing artifacts due to undersampling. Thus, the CBCT taken during each fraction could not be used directly for evaluation of dosimetry [15].

While CBCT HU are known to be inaccurate, regions of extremely low attenuation such as bowel gas are relatively clearly defined. Thus, we roughly segmented the air region using HU-based thresholding with a threshold of −500 HU. These contours were manually reviewed and modified to account for noise and other CBCT artifacts. The contours of bowel gas were also reviewed and confirmed by a radiation oncologist.

We then propagated the delineated air from the CBCT to the pCT using rigid image registration. The rigid image registration was performed by matching the bony configuration observed for each CBCT to the pCT. Once the bowel air of CBCT was copied to the pCT, the voxels of pCT bowel air which did not overlap with the CBCT of the bowel air were assigned a value with an RMD of 1.0 (with soft tissue used as reference). This is identical to the pattern of replacement used in pattern 1 and the first pattern in Kusano et al., when it was assumed that the region held no gas. Meanwhile, the voxels of CBCT bowel air were assigned a value of 0.001 g/cm^3^ (or −1000 HU), which corresponds to the RMD of air. In some cases, the gas volume defined for the CBCT lay outside of the body delineated for the pCT due to body contour variations. In these cases, the volume lying outside the body was discarded. No overlap was found between the gas pockets of the CBCT and the CTV. The image made by registering the air of the CBCT to the pCT was called the fractional CT (fCT) [16]. By using the fCT, we expected to be able to evaluate the isolated effects of only gastrointestinal gas, without the effects of other anatomical variations (such as interfractional tumor movement, body contour variation).

Using the fCT, the dose was calculated in two ways. In the first method, the dose was evaluated for every available fCT, for every individual beam direction (anterior, posterior, oblique, lateral), and for each pattern. Thus, a total of 640 (8 × 5 × 4 × 4; 8 for every patient, 5 for every CBCT image, 4 for every beam direction, and 4 for each pattern) dosimetry patterns were analyzed.

In the second method, the dose was summed across different fractions to obtain an accumulated dose. As SABR is typically done in 5 fractions of 50 Gy, there were 5 images available. By contrast, CIRT of LAPC is typically performed in 12 fractions. To account for this, we only used the CBCT images of the first three fractions. Each CBCT image was irradiated from the anterior, posterior, posterior oblique, and lateral direction, with each beam in 1 fraction at a dose of 4.6 GyE. Thus, a single beam corresponded to a single fraction of treatment. The fractional doses applied to the fCT were accumulated deformably. Cumulative doses better reflect actual tumor coverage and OAR radiation exposure in clinical practice. A total of 32 (8 × 4) dosimetry patterns were analyzed. Due to the paucity of data, less statistically significant results were found.

### Quantitative evaluation

To quantify the bowel gas variation between each fraction and the planning CT, we used the symmetric variation Eq. 1, which is consistent with the technique used by Yao et al. while examining the effects of rectal gas on proton therapy of prostate cancer [17]:

1 $$\begin{aligned}{}&\Delta V_{\mathrm{air}}=\left(V_{\mathrm{air}}^{\mathrm{pCT}}\cup V_{\mathrm{air}}^{\mathrm{CBCT}}\right)\backslash \left(V_{\mathrm{air}}^{\mathrm{pCT}}\cap V_{\mathrm{air}}^{\mathrm{CBCT}}\right)=\\&V_{\mathrm{air}}^{\mathrm{pCT}}\oplus V_{\mathrm{air}}^{\mathrm{CBCT}}\end{aligned}$$

However, as bowel air delineation had been done across the longitudinal length of the planning tumor volume, the effect of tumor size had an evident impact on bowel gas variation. Thus, we normalized the symmetric variation by dividing it by the size of longitudinal length, as shown in Eq. 2. Conceptually, this represents the average area of bowel air for each CT slice and is thus not confounded by the size of the tumor.

2 $$\overline{\Delta V_{\mathrm{air}}}=\frac{V_{\mathrm{air}}^{\mathrm{pCT}}\oplus V_{\mathrm{air}}^{\mathrm{CBCT}}}{\text{Longitudinal Length of PTV}}$$

To quantify the dosimetric variation shown between each patient, we examined the CTV and PTV coverage which were given by *V*_95*%*_, or the relative area covered by 95% of the prescribed dose.

We also examined the homogeneity index (HI) for the CTV:

3 $$HI=\frac{D_{2\mathrm{{\%}}}-D_{98\mathrm{{\%}}}}{D_{50{\%}}}$$

$D_{2\mathrm{{\%}}}$ indicates the minimum dose received by the most irradiated 2% of the CTV, $D_{98\mathrm{{\%}}}$ indicates the minimum dose received by at least 98% of the CTV, and *D*_50*%*_ represents the median dose received by the CTV. We also evaluated the dose to the OAR. Specifically, we examined the $D_{2\mathrm{cc}}$ to the GI tract (which included the stomach, duodenum, jejunum, small intestine, and bowel) as well as the $D_{\max }$ to the spinal cord.

### Statistical analysis

To evaluate the correlation between variations in G‑gas obtained as $\overline{\Delta V_{\mathrm{air}}}$ and the dose to the target volume or the OAR (*V*_95*%*_, HI for the target volume, $D_{2\mathrm{cc}}$ for the GI tract, and $D_{\max }$ for the spinal cord), we linearly fitted the data using the least squares method. The significance of the correlation was judged using the student’s *t*-test for the Pearson correlation coefficient.

The difference between the three patterns of replacement and the pattern with no replacement were evaluated using the Quade test, as there was judged to be no normality for the dataset based on the Shapiro–Wilk test. Furthermore, the dependent variable was a continuous quantitative variable within a bounded ratio, and four independent patterns needed to be evaluated. The Quade test and the Friedman test are both nonparametric tests that analyze paired, dependent samples. However, a Quade test is known to be more robust than a Friedman test when there are less than five independent patterns being evaluated [18]. If a Quade test detected a significant difference, a Wilcoxon signed-rank test with Bonferroni correction was performed for post hoc analysis. The significance level of all statistical tests was taken to be *p* < 0.05. We used R‑4.3.2 (R Foundation for Statistical Computing, Vienna, Austria) for our statistical analysis [19].

**Fig. 2:**
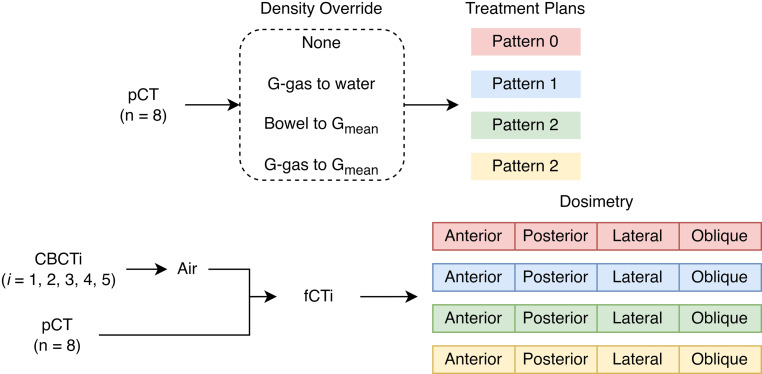
Schematic of the study process. Four different treatment plans were established for each patient based on relative mass density override. Fractional CT images (fCT) were constructed, where the bowel air from each cone-beam computed tomography (CBCT) was registered to the planning CT (pCT). The resulting dose distributions were calculated for each treatment plan and each beam angle. Dose patterns for the first three fCT images for each beam angle were accumulated to simulate tumor coverage and organ at risk radiation exposure for a locally advance pancreatic cancer patient undergoing 12 fractions of 4.6 GyE carbon ion radiotherapy

To summarize, we replaced the material density of selected regions of interest using the replacement patterns discussed above. Based on these modified CT images, we used the RayStation TPS to obtain different irradiation templates. These templates were evaluated on both the original planning CT (without replacement) and the pCT with air propagated from CBCT images or fCT. The evaluation was analyzed in two ways. The first way was to analyze individually for each beam direction and each fCT, as shown in Fig. 2. The second method was to deformably accumulate the dose for the first three fractions of fCT on each beam direction.

## Results

### Bowel air variability correlation with homogeneity index

**Fig. 3:**
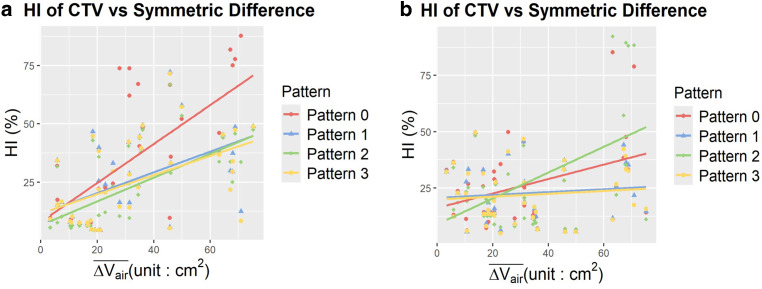
Linear relationships and scatter plots of the homogeneity index (HI) of the clinical target volume (CTV) for **a** the anterior beam and **b** the lateral beam versus the average area for the symmetric difference of air ($\overline{\Delta V_{\mathrm{air}}})$. Data for the posterior and oblique beam are not shown as their linear relationships were not significant

A significant positive linear correlation was observed between bowel air variability, calculated in terms of the symmetric difference defined in Eq. 2, and the HI for the anterior and lateral beam. The scatter plot and linear fitting for HI of the CTV are shown in Fig. 3 for the anterior and lateral beams for all 4 patterns. Data for the posterior and oblique beams are not shown, as no significant correlations were observed for those directions.

The HI of the CTV for the anterior beam demonstrated a significant positive linear relationship with the $\overline{\Delta V_{\mathrm{air}}}$ of the anterior beam. Notably, of the four patterns, pattern 0 had the greatest slope, indicating the greatest amount of HI degradation due to bowel air variation. A different trend was observed for the HI and $\overline{\Delta V_{\mathrm{air}}}$ of the lateral beam. Of the four patterns, pattern 2 had the greatest slope, followed by pattern 0.

### Differences between override patterns

#### Target coverage

**Fig. 4:**
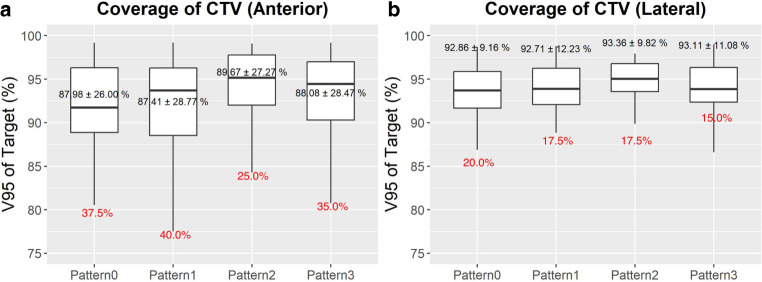
Box and whisker plots for **a** coverage of the clinical target volume (CTV) for the anterior beam, **b** coverage of the CTV for the lateral beam. The best coverage results in terms of the mean coverage (numbers in black) were observed in pattern 2, followed by pattern 3 for both the anterior and lateral beams. Meanwhile, the proportion of patients where coverage decreased by more than 5% (numbers in red) was lowest for pattern 2 for the anterior beam and for pattern 3 for the lateral beam. The coverage plots for the posterior and oblique beams are not shown, as their results were not significant

Average tumor coverage (in terms of *V*_95*%*_) of the CTV was greatest for pattern 2 (89.67%) followed by pattern 3 (88.08%) for the anterior beam (Table 3; Fig. 4). Although the difference in mean coverage shown in Table 3 might appear to be minimal, a deviation of less than ±5% from the treatment plan is desirable in terms of target coverage [9]. This proportion where CTV coverage declined by more than 5% compared to the initial treatment plan was smallest for pattern 2 and pattern 3.

**Table 3:** Average $\overline{V}_{95{\%}}$ (%) for the CTV and PTV

	$\overline{V}_{95{\%}}$* (%) for CTV*				$\overline{V}_{95{\%}}$* (%) for PTV*			
	Pattern 0	Pattern 1	Pattern 2	Pattern 3	Pattern 0	Pattern 1	Pattern 2	Pattern 3
Ant	87.98	87.41	89.67	88.08	84.20	84.09	85.31	84.74
Lat	92.86	92.71	93.36	93.11	90.44	90.47	90.34	90.78
Post	99.37	99.38	99.44	99.41	98.80	98.79	98.80	98.79
Obl	99.54	99.55	99.54	99.53	99.20	99.23	99.16	99.18
Acu	91.68	92.40	92.98	92.87	87.86	89.19	88.11	89.52

*Ant* anterior, *Lat* lateral, *Post* posterior, *Obl* oblique, *Acu* accumulated dose, *CTV* clinical target volume, *PTV* planning target volume

A similar pattern was observed for the lateral beam coverage of the CTV, where pattern 2 and pattern 3 showed the greatest amount of coverage. The proportion of fractions where the CTV coverage declined by more than 5% compared to the original treatment plan was the smallest for pattern 3 at 15.0%. Significant results were not found for the posterior or oblique beams.

Table 3 shows the coverage of the accumulated dose to the CTV. Again, the greatest tumor coverage was shown for pattern 2 followed by pattern 3 for the CTV, although this result was not statistically significant.

#### Homogeneity index

**Fig. 5:**
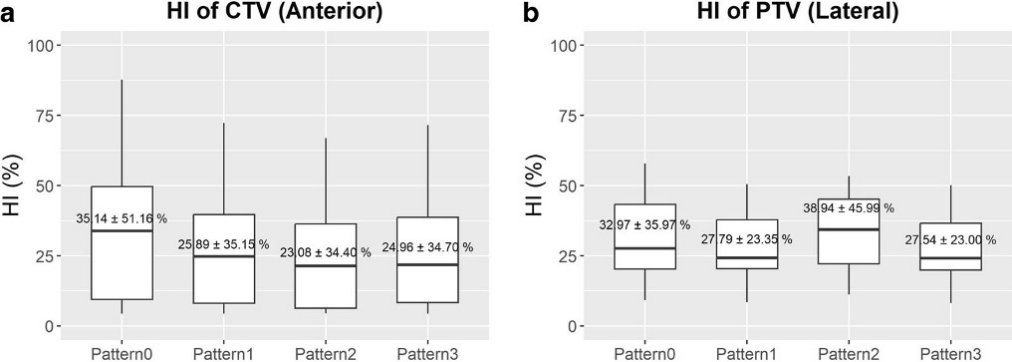
Box and whisker plots for **a** the homogeneity index (HI) of the anterior beam for the clinical target volume (CTV) and **b** HI of the lateral beam for the planning target volume (PTV). The numbers in the middle signify the mean HI and their 95% confidence interval observed for each pattern

Figure 5 and Table 4 show the HI for the anterior beam for the CTV and the HI of the posterior beam for the PTV. The HI for the anterior beam was worst for pattern 0 ($\overline{HI}=35.14{\%}$), while it was best for pattern 2 ($\overline{HI}=23.08{\%}$) followed by pattern 3 ($\overline{HI}=24.96{\%}$) for the anterior beam. While no significant difference was found for the lateral beam, it is notable that pattern 2 demonstrated the worst lateral homogeneity ($\overline{HI}=38.94{\%}$), while pattern 3 showed the best homogeneity ($\overline{HI}=27.54{\%}$).

**Table 4:** Average HI (%) for the CTV and the PTV

	*HI* *(%) for the CTV*				*HI* *(%) for the PTV*			
	Pattern 0	Pattern 1	Pattern 2	Pattern 3	Pattern 0	Pattern 1	Pattern 2	Pattern 3
Ant	35.14	25.89	23.08	24.96	45.74	33.64	38.94	33.60
Lat	26.60	22.64	27.59	21.54	32.54	27.79	38.94	27.54
Post	5.13	5.01	4.65	4.60	5.82	5.75	5.40	5.41
Obl	4.26	4.25	4.23	4.31	4.91	4.87	5.08	5.11
Acu	11.19	7.90	9.05	7.68	13.95	10.28	13.41	10.25

*HI* Homogeneity index

#### Dose to organs at risk

**Fig. 6:**
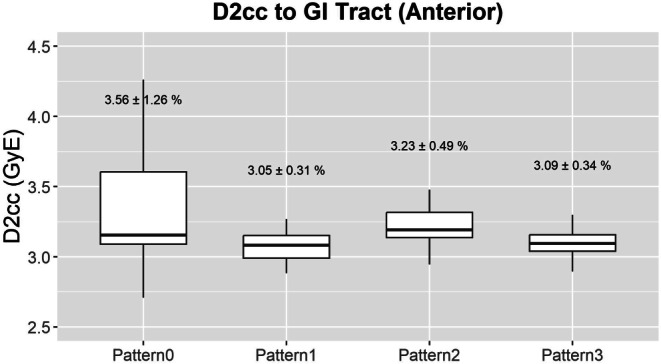
Box and whisker plots for the dose to the gastrointestinal tract in terms of $D_{2\mathrm{cc}}$. The numbers in the middle signify the mean homogeneity index and the 95% confidence interval observed for each pattern

Figure 6 and Table 5 show the *D*_2cc_ of the GI tract and the $D_{\max }$ of the spinal cord for the anterior beam. For the anterior beam, the GI tract received a significantly greater dose per fraction in pattern 0, followed by pattern 2. Pattern 1 and pattern 3 were generally more favorable in terms of their dose to the GI tract. No significant difference was observed for the posterior, oblique, or lateral beams.

**Table 5:** Average $\overline{D_{2\mathrm{cc}}}$ (GyE) for the gastrointestinal (GI) tract

	$\overline{D_{2\mathrm{cc}}}$* (GyE) for the GI tract*			
	Pattern 0	Pattern 1	Pattern 2	Pattern 3
Ant	3.56	3.05	3.23	3.09
Lat	3.46	3.47	3.51	3.45
Post	1.74	1.76	1.75	1.77
Obl	1.73	1.74	1.73	1.79
Acu	23.47	23.42	23.11	23.47

## Discussion

### Dosimetric effects of density-override patterns

This study evaluated density-override patterns for robust optimization against bowel gas variation using CBCT data from patients treated with SABR. We examined the correlation between bowel air variation and CTV homogeneity index (HI). The strongest positive correlation was observed in pattern 0, indicating greater HI degradation with air variation. We further compared each pattern of replacement in terms of HI, coverage of the CTV, and the D_2cc_ of the GI tract. Pattern 0 consistently resulted in higher OAR doses compared to override patterns. These findings reinforce the need for density-override planning, as used in patterns 1–3. This aligns with Kusano et al., who advocate incorporating bowel air into treatment planning via density overrides [13].

Among the override strategies, pattern 2 yielded the highest mean CTV and PTV coverage. This is consistent with the results observed by Kusano et al., which found that the mean gas replacement condition is most favorable in terms of coverage [13]. Pattern 3 consistently ranked second for anterior and lateral beam coverage.

However, pattern 2 delivered a relatively higher GI dose for the anterior beam, ranking just above pattern 0. Although all included patients had GTVs with a distance of at least 5 mm from the bowel wall, patients with closer proximity may face higher bowel toxicity with pattern 2. It was also notable that pattern 2 showed the least favorable homogeneity of the PTV for the lateral beam. This contrasts with Kusano et al., who reported pattern 2 to be the most homogeneous due to minimal HI variation (∆HI) from the original plan [13]. In contrast, our findings suggest that pattern 2 yields higher absolute HI values, indicating poorer homogeneity.

Thus, pattern 2 demonstrated the best average CTV and PTV coverage but had trade-offs: for anterior beams, it resulted in elevated GI doses, ranking just above pattern 0. Additionally, it yielded the least favorable lateral beam homogeneity (highest HI). These discrepancies may reflect anatomical or beam configuration differences between the cohorts and underscore the need for individualized planning based on proximity to the bowel. In this context, pattern 3 emerges as a promising alternative—offering more balanced dosimetric performance with consistently moderate coverage and improved homogeneity, particularly in anatomically sensitive cases.

### Clinical applicability and workflow considerations

As noted earlier, pattern 2 closely resembles Kusano et al.’s second replacement strategy. However, the method proposed by Kusano et al. involved separating the GI tract into the upper–middle stomach, lower stomach, first–second part of the duodenum, third part of the duodenum, fourth part of the duodenum, small intestine, right colon, and left colon [13]. The intestines are mobile and challenging to delineate accurately and are subject to substantial interobserver variability in clinical practice. Thus, pattern 2 took the average HU of the entire bowel without subdivisions, while pattern 3 applied overrides only to identified bowel air regions. Both patterns 2 and 3 extended the delineated OAR, a standard step in our SABR contouring workflow.

While pattern 0 consistently led to higher OAR doses and greater HI degradation, the differences in CTV coverage between override patterns (1–3) and pattern 0 were not always statistically significant. This suggests that although override strategies yield improvements in dosimetric metrics, the clinical magnitude of these improvements—especially in target coverage—may be modest.

However, their contribution to reproducibility and robustness provides clinically meaningful advantages in terms of minimizing treatment uncertainty. Override patterns—especially patterns 2 and 3—showed reduced interpatient and interfractional variability. This is evidenced by a lower proportion of people with more than 5% degradation in coverage and narrower interquartile ranges in coverage and GI dose (Figs. 4 and 6). This reduction in variability suggests that density override not only improves average dosimetric outcomes but also enhances interfractional and interpatient robustness. The consistency afforded by override strategies may help to reduce unexpected CTV underdosage or OAR overdose in daily practice, thus improving reliability.

Bowel gas displacement produces clinically significant perturbations of the planned dose distribution. Although density-override strategies (patterns 1–3) partially mitigate these dosimetric degradations, residual undercoverage persists, demonstrating that planning-stage overrides alone cannot fully compensate for interfractional anatomical variability. These results underscore the importance of stringent bowel gas management protocols (e.g., fasting). Furthermore, adaptive radiotherapy via online replanning or beam angle optimization as proposed by Kawashima et al. may offer additional robustness [20]. We note that our method of density override in the planning stage can naturally incorporate additional angles, as bowel air delineation is not dependent on beam direction.

### Methods for quantifying bowel air variation

There are three different methods for quantifying the variation in bowel air: (a) using the symmetric difference of air using Eq. 1; [17], (b) using the volumetric difference of air $V_{\mathrm{air}}^{\mathrm{pCT}}-V_{\mathrm{air}}^{\mathrm{CBCT}}$ [13, 21], or (c) taking the average symmetric difference (as in our study) using Eq. 2. It was expected that method (c) would be the most successful in explaining the dosimetric variations. A significant bowel movement may be overlooked using method (b), as even though bowel air might have the same volume, it might lie in a different configuration.

Based on the patient data gathered, the method for quantifying the variation in air quality was selected based on the method that showed the greatest number of statistically significant linear relationships and the strength of those relationships, the average R^2^. All planning occurred without replacement, and eight dosimetric indices were analyzed for their linear relationships: *V*_95*%*_, $D_{98\mathrm{{\%}}}$, and *HI* of the CTV and PTV, *D*_2cc_ of the OAR, and *D*_*max*_ of the spinal cord. The number of significant relationships and the average R^2^ values are shown in Table 6. The number of significant relationships shown was 20, 20, and 17 for methods (a), (b), and (c), respectively. The average R^2^ values for all beams combined were 0.239, 0.201, and 0.270 for methods (a), (b) and (c), respectively.

**Table 6:** Number of significant linear relationships shown and the average R^2^ for the significant linear relationships based on the method of quantifying volumetric difference

	Method	# of Sig Rel	Average R^2^
Anterior	(a)	7	0.340
	(b)	6	0.283
	(c)	7	0.410
Lateral	(a)	7	0.231
	(b)	4	0.142
	(c)	7	0.182
Oblique	(a)	5	0.116
	(b)	6	0.193
	(c)	2	0.145
Posterior	(a)	1	0.196
	(b)	4	0.147
	(c)	1	0.164

Even though it showed the lowest number of significant linear relationships, the greatest average R^2^ was shown for method (c). Furthermore, the anterior or lateral beam (c) showed a greater number of significant relationships when compared to (b). Thus, method (c) was selected to quantify the variation in air quality within our study.

# of Sig Rel number of significant relationships, as judged by *p* < 0.05

### Limitations

Our study is limited by the small cohort size (*n* = 8), which restricts the statistical power for some endpoints. Additionally, only five CBCT images were available per patient, making it impossible to simulate all 12 fractions of CIRT treatment to evaluate the cumulative dose. Instead, the first three CBCTs were used, with each CBCT image irradiated from each direction at one fraction. Despite this limitation, the consistency of pattern-specific trends across beams and dosimetric metrics suggests that the findings are not merely incidental. Future prospective studies with larger patient numbers and multi-institutional data are needed to validate these trends and develop standardized override protocols tailored to tumor location and gas distribution patterns.

## Conclusion

This study demonstrated that treatment plans for which density override was performed were more robust against gastrointestinal gas variation compared to treatment plans for which density override was not performed, especially in terms of the dose to OAR. Furthermore, out of multiple density-override patterns, pattern 2 was found to be most robust in terms of target coverage, followed by pattern 3. However, pattern 2 was shown to have poor homogeneity and be unfavorable in terms of the dose to the GI tract. Thus, if it becomes desirable to uniformly deliver a high dose to the target volume, or if the target is adjacent to the GI tract, pattern 3 should be considered.

## Data Availability

The data that support the findings of this study are available from the corresponding author upon reasonable request.
